# Integrated Bioinformatics and Experimental Approaches Identified the Role of NPPA in the Proliferation and the Malignant Behavior of Breast Cancer

**DOI:** 10.1155/2021/7876489

**Published:** 2021-09-27

**Authors:** Aijun Sun, Xiaonan Sheng, Jinhai Tang, Zhenfeng Yu, Jian Zhang

**Affiliations:** ^1^The First Clinical Medical College, Nanjing University of Chinese Medicine, Xianlin Road 138, Nanjing 210023, China; ^2^Department of Thyroid and Breast Oncological Surgery, Xuzhou Medical College Affiliated Huaian Hospital, Huaihai South Road 62#, Huaian, Jiangsu 223001, China; ^3^Renji Hospital, School of Medicine, Shanghai Jiao Tong University, No. 1630 Dongfang Shanghai 200127, China; ^4^Department of General Surgery, The First Affiliated Hospital with Nanjing Medical University, Nanjing 210029, China; ^5^Department of General Surgery, Shanghai Fengxian Central Hospital, 6600 NanFeng Road, 201499, China

## Abstract

Breast cancer is the 3^rd^ most common type of malignant tumor worldwide with high heterogeneity, frequent recurrence, and high metastasis tendency. In this study, we aimed to demonstrate the value of extracellular matrix- (ECM-) related genes in breast cancer patients. The overall expression of ECM is assessed with a novel SC3 clustering method, and patients were divided into two clusters with diverse recurrence rate. We established the Cox regression model in breast cancer patients and identified NPPA as an independent prognostic marker. The NPPA expression is downregulated in breast cancer patients, independent of the ER status, PR status, stemness score, and immune infiltrating condition. And we observed the enhanced proliferation, migration, and invasion potential of breast cancer cells after NPPA depletion. Further, we predicted the transcription modulation of NPPA with PROMO and JASPAR. And we further validated the binding of MZF1 to the -318 bp~-452 bp region of the NPPA promoter with chromatin immunoprecipitation and dual luciferase assay. Together, our study identified NPPA as a potential prognostic biomarker for breast cancer patients, whose downregulation is associated with an enhanced malignant behavior of breast cancer cells both in vivo and in vitro and identified the transcription regulation of NPPA by MZF1.

## 1. Introduction

Breast cancer is among the most common types of malignant tumor worldwide and the most common type of malignant tumor in women, accounting for at least half million of death annually [1]. Breast cancer typically has a high level of heterogeneity and can be further categorized into multiple subtypes with respect to various molecular and pathological signatures, including Her2 status, estrogen receptor (ER) status, progesterone receptor (PR) status, and PAM50 features [2, 3]. Like other types of malignant tumors, early diagnosed breast cancer are largely curable with the combination of primary surgical approaches and multiple following effective subsequential therapies, including chemotherapy, radiotherapy, or targeted therapy [4–6]. However, the high recurrence rate of breast cancer remains a critical barrier for the long-term survival of patients. Also, the underlying mechanisms of recurrence and distant metastasis are not fully investigated.

Extracellular matrix (ECM) typically includes fibrillar collagens, fibronectin, specific laminins, proteoglycans, and matricellular proteins [7]. The extracellular matrix-related genes comprise of at least 555 proteins and are considered to have a critical influence on the prognosis of cancer [8], whose alteration are closely linked to the invasion and metastasis of the tumor [9–11], such as brain and lung metastases [12]. The chemical and biological properties of ECM are highly complicated and are delicately modulated in the tumor microenvironment, including local hypoxia, nutrition deprivation, and the infiltration of immune cells [13–17]. The modeling of cancer ECM by various cell types, such as epithelia and stroma, has been proposed to have a profound effect on the progression of cancer [18]. While many of the ECM-related genes and their regulation network have been identified [19–21], the ECM-related genes in the Gene Ontology (GO) database comprises of as many as 555 genes, whose biological functions are largely unknown. Besides, the approaches to modulate ECM in breast cancer tissues are very limited [8, 21, 22].

NPPA (natriuretic peptide precursor A) belongs to the ECM genes in the GO database, whose expression is considered to involve in the familial atrial fibrillation, heart development, and hypertension [23–25]. However, the function and expression of NPPA in cancer, such as breast cancer, have not been explored.

The single-cell consensus matrix (SC3) model was proposed by Kiselev et al. as a quick and efficient nonmonitored clustering method, primarily used in the analysis of single-cell sequencing data [26]. With this method, we could calculate the eigenvector of each sample with all the expression genes and effectively distinguish the subclusters of breast cancer patients while identifying the potential markers for each cluster.

Here, in this study, we have analyzed the expression of all 555 ECM-related genes in the TCGA breast cancer database and performed a novel SC3 method to cluster breast cancer patients. We have identified a 49-gene ECM signature in breast cancer patients and eventually identified NPPA as an independent prognostic marker with the forward stepwise multivariate Cox regression model. Further, we validated the proliferation suppression function of NPPA both in vivo and in vitro. Lastly, we predicted and validated the transcription modulation of NPPA by MZF1, at the -318~-452 bp of the NPPA promoter. Together, our findings identified NPPA as a prognostic marker, unveiled the biological function of NPPA, and explored the transcription modulation of NPPA by MZF1, which we believe would expand the horizon for breast cancer treatment.

## 2. Material and Methods

### 2.1. Data Collection

mRNA sequencing data, molecular categories, immunohistochemistry (IHC) staining data, and clinical information of breast cancer patients and other cancer types were obtained from the TCGA BRCA database (https://tcga-data.nci.nih.gov/), Human Protein Atlas (https://www.proteinatlas.org/), IST Online (https://ist.medisapiens.com/), GEO database (https://www.ncbi.nlm.nih.gov/), and GEPIA (http://gepia.cancer-pku.cn/index.html), respectively. The expression pattern of normal breast tissues and paratumor tissues was acquired from the TCGA database, GEO database (GES65261), and GTEx database (https://xenabrowser.net/), respectively.

Stemness score and ssGSEA result from mRNA were acquired from the TCGA PAN-CANCER database (https://xenabrowser.net/datapages/). Levels of different immune cells in breast cancer patients were acquired from GEPIA (http://gepia.cancer-pku.cn/index.html).

Telomere length for breast cancer patients was acquired from previous research [27].

### 2.2. Kaplan-Meier Analysis

Kaplan-Meier analysis was performed with GraphPad (https://www.graphpad.com/), SPSS (https://www.ibm.com/products/spss-statistics), and GEPIA to calculate log-rank significance in different groups of breast cancer patients. Besides, Kaplan-Meier analysis was performed with SPSS and visualized with GraphPad to stratify patients with respect to ER status, PR status, postoperation radiation, and PAM50 subtypes in breast cancer patients.

The overall survival (OS), disease-specific survival (DSS), disease-free interval (DFI), and progression-free interval (PFI) were compared in breast cancer patients.

### 2.3. Multiple Variate Cox Regression

Multiple variate Cox regression was performed with SPSS. The forward stepwise method was performed with the threshold of *p* < 0.05 to include and *p* > 0.10 to acquire independent factors for the prognosis of breast cancer patients.

Significant and independent factors predicting OS, DSS, DFI, or PFI were selected in breast cancer patients. The corresponding risk scores for OS, DSS, DFI, and PFI were calculated and assessed, respectively.

### 2.4. Heatmap and Hierarchical Clustering

Heatmap and hierarchical clustering were performed with the MeV software (https://sourceforge.net/projects/mev-tm4/). Expression levels of all genes included in the analysis were normalized with respect to the median of the gene across all patients, and the color scale was normalized and set to -2~2. Hierarchical clustering was performed with the Euclidean clustering method with average linkage and optimized gene/sample order.

Heatmap and the following hierarchical clustering enabled us to directly visualize the expression pattern of certain clusters of genes and samples.

### 2.5. Data Analysis

IHC data was analyzed with Image-Pro Plus (https://www.totalsmart.com.tw/cn/image-pro-plus).

The correlation between two groups of samples was performed and plotted with GraphPad. Linear regression was performed with SPSS, and 95% CI was plotted in a dotted line.

Student's *t*-test was performed with GraphPad.

Single-cell consensus clustering (SC3) was carried out with R package [26], and the dimension reduction method with the t-SNE method was archived with python [28].

### 2.6. Statistical Analysis

Student's *t-*test was performed to compare the expression level of different groups unless otherwise stated. Paired Student's *t*-test was used to compare the NPPA and MZF1 mRNA levels in tumor tissues and paired paratumor tissues.

Log-rank analysis was utilized to compare the survival of breast cancer patients. Pearson's correlation coefficient was calculated between two genes in breast cancer (such as NPPA and MZF1).

### 2.7. Prediction of the Transcriptional Modulation of NPPA

To further explore the transcriptional modulation of NPPA in breast cancer patients, the DNA sequence of the SMC4 promoter region (-1000 bp~-1 bp) was obtained from the UCSC database (http://www.genome.ucsc.edu/index.html). The binding affinity of all transcription factors to all binding sites were predicted in both the PROMO database (http://alggen.lsi.upc.es/recerca/frame-recerca.html) and JASPAR database (http://jaspar.genereg.net/).

Next, the mutual transcription factors in both database: transcription factors with dissimilarity < 5% in the PROMO database, or with relative score > 90% in the JASPAR database, were selected as potential transcription factors for the modulation of NPPA. Lastly, we analyzed the expression level of NPPA and candidate transcription factors and transcription factors in the TCGA database and transcription factors with Pearson's *R* > 0.25 or <-0.25 were selected for further validation.

### 2.8. Chromatin Immunoprecipitation (ChIP) and Dual Luciferase Reporter Assay

ChIP experiment was performed with the Chromatin Immunoprecipitation kit (Merck Millipore, MA, USA) according to the manufacturer's instructions. Quantitative PCR was used to measure the relative enrichment of MZF1 on the NPPA promoter, using primers specific for each target gene promoter. Primer sequences were as follows: primer for -318~-452, F: 5′- GCTGGCTGCCTGCCATTTCCTC -3′, R: 5′- CGTGCCTCAGGATTCTTTC -3′ and primer for -889~-762, F: 5′- TCCTCCATCGGTCAAGTTGC-3′, R: 5′- CGACCCTCCTCCAGCATGCT-3′.

Dual luciferase reporter assay was performed with firefly plasmid carrying -300 bp~-500 bp of the NPPA promoter and control Renilla plasmid. 10 : 1 of firefly and Renilla plasmid was cotransfected to breast cancer cell lines. After 2 days of transfection, cells were harvested and lysed in lysis buffer (Promega, Madison, WI, USA), and the activity of both plasmid was detected by the Dual-Luciferase Reporter Assay System (Promega). The results were normalized to the Renilla activities and analyzed with GraphPad.

### 2.9. Constructs and Transfections

Specific target shRNAs and a nontarget shRNA were cloned into lentiviral vector pLKO.1. 2 different shRNA sequences were employed in this study: shNPPA seq.1: 5′- GAGCTAATCCCATGTACAATG-3′ and shNPPA seq.2: 5′- TTGTACATGGGATTAGCTCTG-3′.

The overexpression of flag-tagged MZF1 protein and shRNA transfection were achieved with plasmid as previously reported [29].

### 2.10. Cell Culture

Breast cancer cells MCF-7, MDA-MB-231, BT-20, and HCC1937 are obtained from Shanghai Institute of Oncology. MCF-7 and MDA-MB-231 cells were cultured in DMEM/F12 supplemented with 10% FBS and 1% P/S. BT-20 cells were cultured in MEM supplemented with 10% FBS and 1% P/S. HCC1937 were cultured in the RPMI-1640 medium supplemented with 10% FBS and 1% P/S.

All cells were cultured in a humidified condition at 37 degrees with 5% CO_2_.

### 2.11. Proliferation Assay of Breast Cancer Cells

For the proliferation assay of 4 breast cancer cell lines, 2000 cells were seeded in triplicates in a 96-well plate on the first day. Cell viability was measured for 5 consecutive days with 1 hour incubation in the Cell Counting Kit-8 (MedChemExpress).

### 2.12. Invasion and Migration Analysis of Breast Cancer Cells

Matrigel invasion assay was performed as previously reported [30]. In brief, Matrigel was dissolved at 4 degrees, diluted to 25% with DMEM, and coated on top of the Transwell (25 *μ*l per Transwell). A complete medium was added to the lower chamber of the 24-well plate, and cells were resuspended in DMEM at 5^∗^10^5^/ml and 100 *μ*l of the cells seeded on top of the Matrigel. After 24~48 h, cells were fixed and stained with crystal violet.

Migration assay was also performed as previously reported [31]. In brief, a complete medium was added to the lower chamber of the 24-well plate. Cells were then resuspended in DMEM at 5^∗^10^5^/ml, and 100 *μ*l of the cells was seeded on top of the Transwell. After 24~48 h, cells were fixed and stained with crystal violet.

### 2.13. Quantitative PCR (qPCR)

qPCR was performed as previously reported.

Primers used are as follows: NPPA_F: CAACGCAGACCTGATGGATTT; NPPA_R: AGCCCCCGCTTCTTCATTC; MZF1_F: TCCAGGTAGTGTAAGCCCTCA; MZF1_R: TCCTGTTCACTCCTCAGATCG; GAPDH_F: AAGGTCGGAGTCAACGGATT; GAPDH_R: CTCCTGGAAGATGGTGATGG; ACTIN_F: CCTGGCACCCAGCACAAT; and ACTIN_R: GGGCCGGACTCGTCATACT.

### 2.14. In Vivo Experiments

Nude mouse xenograft model was established with the MCF-7 breast cancer cell line. In brief, 2^∗^10^6^ MCF-7 cells transfected with shCtrl, shNPPA seq.1, or shNPPA seq.2, respectively, were injected to 6-8 weeks old nude mice. Tumor volume was measured every 3-4 days once the tumor was observable. Mice were sacrificed once the tumor volume was close to 1000 mm^3^.

## 3. Results

### 3.1. The Landscape of ECM-Related Genes in Breast Cancer Patients

**Figure 1:**
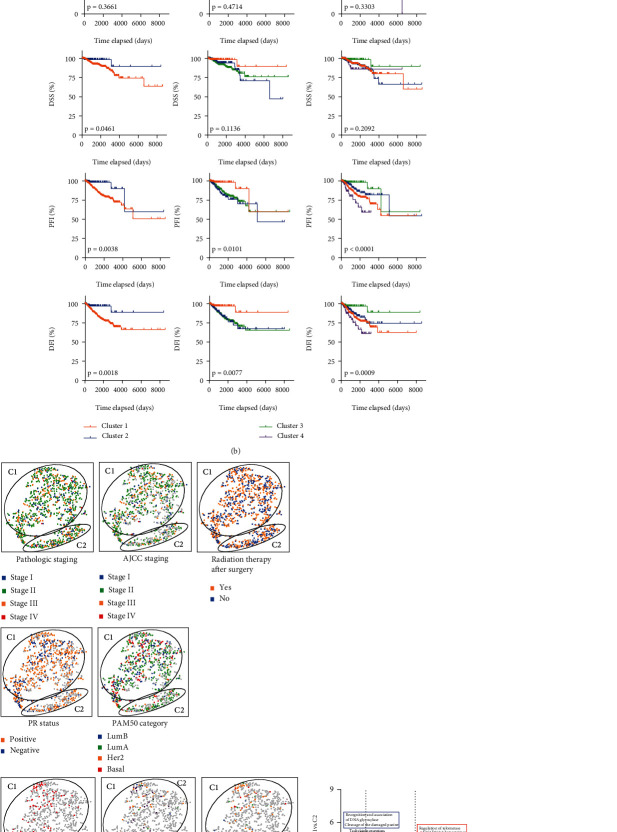
The landscape of ECM-related genes in breast cancer patients. (a) t-SNE method showing the SC3 clustering result. 2 to 4 groups of patients were demonstrated and labeled. (b) Kaplan-Meier analysis comparing the OS, DSS, DFI, and PFI of different clusters of breast cancer patients in (a). (c) The labeling of pathological stages, AJCC stages, radiation therapy condition, PR status, PAM50 subtypes, postoperative new tumor events, new tumor event anatomic site, and new tumor event types in breast cancer patients. (d) Volcano plot showing the −log(*p*) and the difference in ssGSEA results in Cluster 1 and Cluster 2 breast cancer patients. Significant pathways with difference in ssGSEA > 0.25 were labeled in red (enhanced in Cluster 1) or blue (suppressed in Cluster 1).

A total of 568 extracellular matrix- (ECM-) related genes were acquired from a Gene Ontology term, GO_EXTRACELLULAR_MATRIX, in the GSEA database (http://www.gsea-msigdb.org). To interpret the significance of extracellular matrix-related genes in breast cancer patient, we demonstrated the expression matrix of these genes from the TCGA database (https://portal.gdc.cancer.gov/). A nonsupervised clustering method, SC3 clustering method (single-cell consensus matrix), was performed, and the clustering results were visualized with a *t*-distributed stochastic neighbor embedding (t-SNE) method (Figure 1(a)). To determine the appropriate number of clusters, we compared the overall survival (OS), disease-specific survival (DSS), disease-free interval (DFI), and progression-free interval (PFI) with Kaplan-Meier analysis in all clusters of patients. And a significant difference in DSS, DFI, and PFI can only be observed in breast cancer patients when they were divided into 2 clusters (Figure 1(b)).

To illustrate the significance of the clustering, we labeled the pathological staging, AJCC staging, radiation treatment, PR status, PAM50 status, new tumor events status, new tumor anatomic site, and new tumor event types in these 2 clusters of breast cancer patients (Figure 1(c) and Suppl. Figure 1A-1C). As shown in Figure 1(c), there is no difference in the pathological and AJCC staging, postoperation treatment, and the molecular subtypes of the patients (Suppl. Figure 1A-1B), yet we observed a dramatic increase in the relapse of the tumor (Suppl. Figure 1C). Further bioinformatics analysis unveiled that the new tumor events after initial treatment were most commonly distant metastasis, most commonly observed in bones (Suppl. Figure 1C).

With the single-sample gene set enrichment analysis (ssGSEA), we observed the most significantly altered pathways in Cluster 1 and Cluster 2 (Figure 1(d)). And pathways correlated with the tumor recurrence, such as regulation of telomerase pathway, SMAD2/3/4 heterotrimer-regulated transcription pathway and the activation of NIMA kinases pathway, were significantly activated, while the pathways involved in the DNA damage repair pathways, such as recognition and association of DNA glycosylases pathway and the cleavage of the damaged purine pathway, were inhibited.

### 3.2. Identification of NPPA as an Independent Prognostic Marker for Breast Cancer Patients

**Figure 2:**
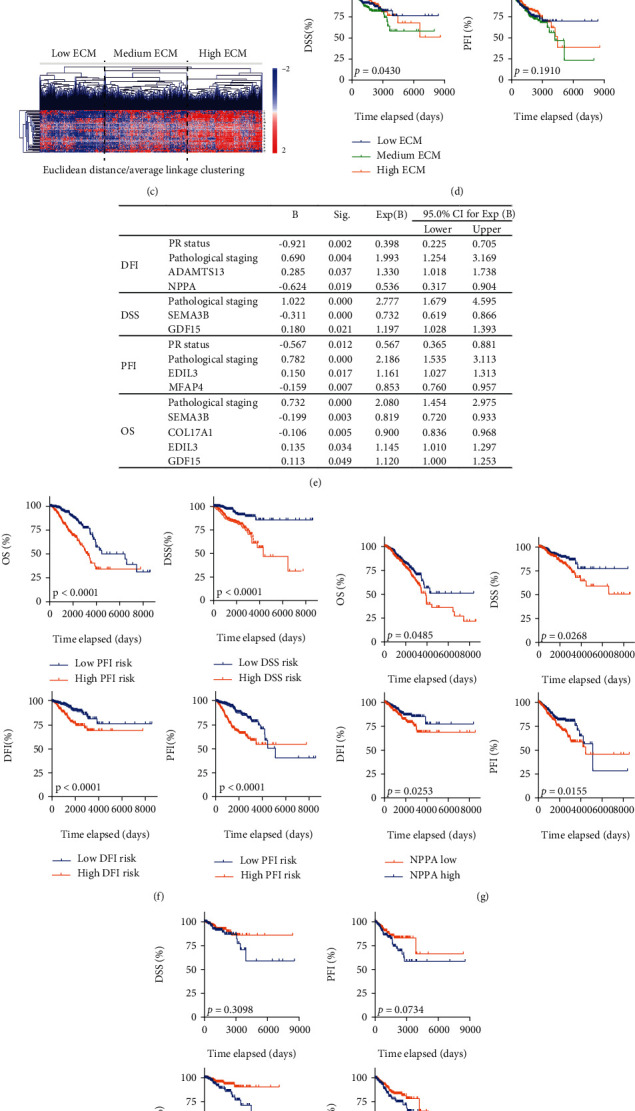
Identification of NPPA as an independent prognostic marker for breast cancer patients. (a) Heatmap showing the marker genes in SC3 clustering results. (b) Volcano plot showing the significance of genes in Cluster 1 vs. Cluster 2 and in SC3 clustering result. Mutually significant genes were defined as marker genes and were labeled in red. (c) Heatmap showing the expression of ECM marker genes in breast cancer patients. Three major clusters of patients were identified as high, medium, and low ECM levels. (d) Kaplan-Meier analysis comparing the DSS and PFI of the high-, medium-, and low-ECM breast cancer patients. (e) Multivariate Cox regression results showing the independent marker genes and clinical features for the prognosis of breast cancer patients. Risk factors for OS, DSS, DFI, and PFI were calculated with respect to the Cox regression results. (f) Kaplan-Meier analysis comparing the high vs. low risk scores in OS, DSS, DFI, and PFI was demonstrated in breast cancer patients. (g, h) Kaplan-Meier analysis comparing the high vs. low NPPA in OS, DSS, DFI, and PFI was demonstrated in breast cancer patients (g) or in breast cancer patients stratified by postoperative radiation therapy.

We collected the most altered genes between Cluster 1 and Cluster 2 as potential markers for breast cancer patients (Figure 2(a)), and the significance between the mRNA level of all ECM genes in Cluster 1 and Cluster 2 patients was calculated. Eventually, 49 marker genes with *p* < 10^−10^ in SC3 method and with *p* < 10^−5^ between Clusters 1 and 2 were further selected as the signature of ECM-related genes (Figure 2(b)).

With hierarchical clustering, breast cancer patients were further categorized into 3 different levels with respect to the expression pattern of ECM marker genes, termed as the low-, medium-, and high-ECM groups, respectively, (Figure 2(c)). Kaplan-Meier analysis showed patients with higher ECM level showed impaired DSS and a tendency to reduce PFI in breast cancer patients (Figure 2(d)).

To further explore the prognostic significance of these marker genes in breast cancer patients, we performed a multivariate Cox regression model to select the independent markers for DFI, DSS, PFI, and OS (Figure 2(e)). Clinical features, including CN clusters, ER status, PAM50 status, PR status, TNM staging, surgical procedure name, histological type, and pathological staging together with 49 ECM markers were included in the model. Results showed that PR status, pathological staging, and the expression of ADAMTS13 and NPPA correlated with the DFI; pathological staging and the expression of SEMA3B and GDF15 correlated with the DSS; PR status, pathological staging, and the expression of EDIL3 and MFAP4 correlated with the PFI; and pathological staging and the expression of SEMA3B, COL17A1, EDIL3, and GDF15 correlated with the OS of the breast cancer patients (Figure 2(e)).

Risk scores for the OS, DSS, DFI, and PFI were calculated with results in Figure 2(e), respectively, and the significance of these risk scores was demonstrated (Figure 2(f)). Then, we assessed the prognostic value of all these independent genes and results showed only SEMA3B and NPPA could significantly predict the DFI, PFI, and DSS of breast cancer patients (Figure 2(g) and Suppl. Figure 2A). Considering the fact that the biological function of SEMA3B has been proposed, we seek to unveil the function of NPPA in breast cancer.

Next, we observed that the NPPA was significantly correlated with the DSS of breast cancer patients stratified by postoperative radiation therapy condition (Figure 2(h)), but not in breast cancer patients stratified by ER status (Suppl. Figure 2B), PR status (Suppl. Figure 2C), and PAM50 subtypes (Suppl. Figure 2D).

### 3.3. The Expression of NPPA Was Impaired in Breast Cancer Tissues

**Figure 3:**
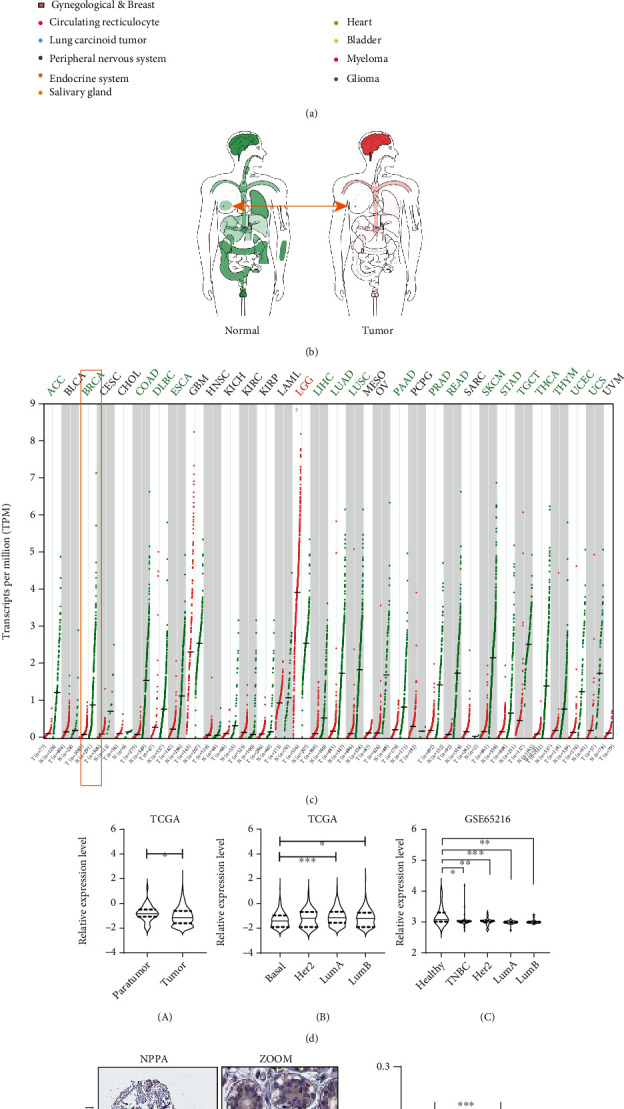
The expression of NPPA was impaired in breast cancer tissues. (a–c) The expression pattern of NPPA in IST Online (a), GEPIA (b), or in TCGA (c). (d) The expression of NPPA mRNA in tumor and paratumor tissues (A), in different PAM50 subtypes (B), and in GSE65216 database (C). (e, f) The expression (e) and quantification (f) of NPPA protein level in the Human Protein Atlas with immunohistochemistry. Average optical density (AOD) was used to determine the protein level of NPPA. For the data in (d), (A) was analyzed with paired Student's *t*-test and the rest were analyzed with Student's *t*-test. ^∗^*p* < 0.05, ^∗∗^*p* < 0.01, and ^∗∗∗^*p* < 0.001.

We compared the expression pattern of NPPA in both normal and malignant tumor tissues with IST Online. As shown in Figure 3(a), NPPA was highly expressed in the normal endocrine system, myeloma lung carcinoid tumor, and glioma, yet NPPA was significantly reduced in breast cancer (Figure 3(b)). Consistently, we observed that the expression of NPPA was suppressed in various tumor tissues and was only overexpressed in lower grade glioma (Figure 3(c)).

Further, we showed NPPA was decreased in breast cancer in the TCGA and GSE65216 databases, especially in the basal group (Figure 3(d)). Next, we collected the NPPA immunohistochemistry (IHC) staining in the normal and breast cancer tissues from the Human Protein Atlas database (Figure 3(e)) and consistently impaired NPPA level were observed in breast tumor tissues (Figure 3(f)).

However, the expression of NPPA was not altered in patients with/without radiation therapy (Suppl. Figure 3A) and in patients with different ER status (Suppl. Figure 3B) or histological staging (Suppl. Figure 3C). Further, we observed a weak correlation of NPPA with stemness score (Suppl. Figure 3D), relative telomere length (Suppl. Figure 3E), and various T cells (Suppl. Figure 3F) in breast cancer patients.

### 3.4. NPPA Silencing Promotes the Malignant Behavior of Breast Cancer Cells

**Figure 4:**
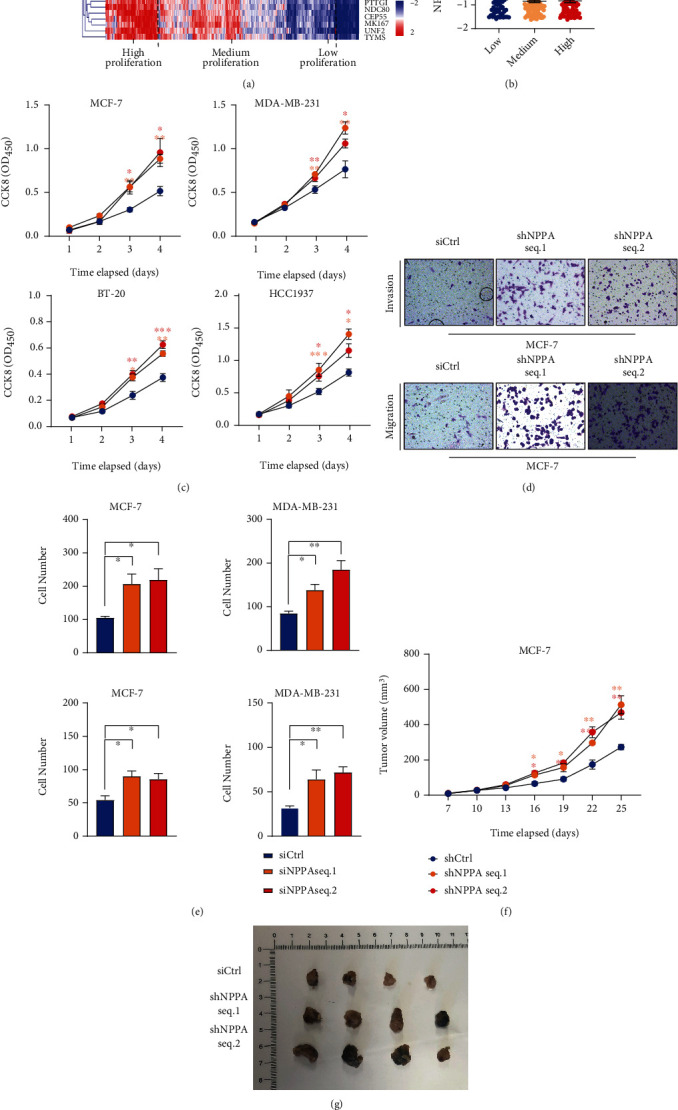
NPPA silencing promotes the malignant behavior of breast cancer cells. (a) Heatmap showing the proliferation potential of breast cancer patients in the TCGA database. The top three clusters of patients were identified as the high, medium, and low proliferation groups. (b) Quantification of NPPA mRNA level in the high, medium, and low proliferation breast cancer patients. (c) The proliferation curve of NPPA depleted or control cells. Experiments were performed in 4 different breast cancer cell lines. (d, e) The typical figure (d) and the quantification (e) of MCF-7 cells and MDA-MB-231 cells in the Transwell experiments. Migration (A) and invasion (B) were assessed, respectively, in NPPA depleted or control cells. (f, g) The proliferation curve (f) and the tumor image (g) of MCF-7 nude mouse xenograft. Tumor growth and tumor volume were assessed in NPPA depleted or control MCF-7 cells. Data are represented as mean ± SEM, ^∗^*p* < 0.05, ^∗∗^*p* < 0.01, and ^∗∗∗^*p* < 0.001. The data were analyzed using Student's *t*-test.

Breast cancer patients were further divided into three different groups according to their separate proliferation potential (Figure 4(a)), and the expression level of NPPA was gradually decreased in patients with higher proliferation potential (Figure 4(b)).

To validate our findings with the bioinformatics analysis, we established 4 different breast cancer cell lines with stably NPPA knockdown with two different sequences of shRNA (Suppl. Figure 4A). The NPPA knockdown with two different shRNAs could significantly enhance the proliferation potential of 4 different breast cancer cells, despite their subtypes or ER status (Figure 4(c)).

To confirm whether NAAP also has a critical role in the migration and the invasion of breast cancer cells, we performed Transwell experiments with MCF-7 and MDA-MB-231 cells. Results showed NPPA knockdown could significantly enhance the migration and the invasion of breast cancer cells (Figures 4(d) and 4(e)). However, the necrosis and the apoptosis of MCF-7 and MDA-MB-231 cells were not affected by NPPA knockdown (Suppl. Figure 4B-4C).

Next, we sought to validate our findings in nude mouse xenograft model. As expected, we observed the enhanced proliferation of NPPA knockdown MCF-7 cells, as compared with the control groups (Figures 4(f) and 4(g) and Suppl. Figure 4D-4E).

### 3.5. NPPA Was Transcriptionally Modulated by MZF1 in Breast Cancer Cells

**Figure 5:**
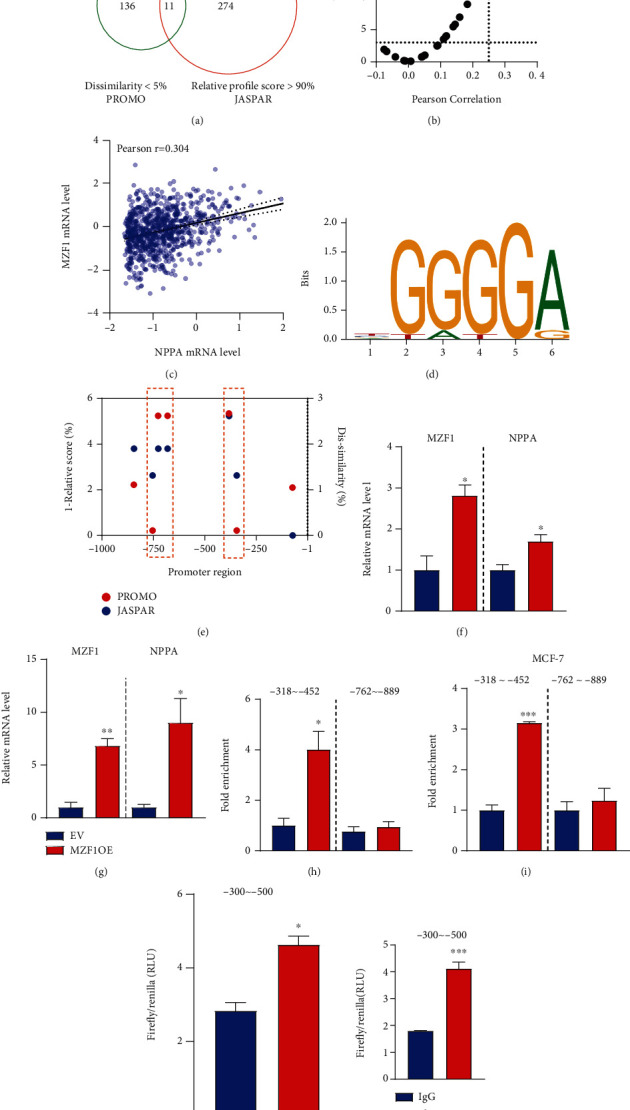
NPPA was transcriptionally modulated by MZF1 in breast cancer cells. (a) Veen plot showing the mutual transcription factors identified by both PROMO and JASPAR. (b) Dot plot showing the Pearson correlation between mRNA level of NPPA and 11 transcription factors. MZF1 was labeled in red. (c) Dot plot showing the correlation between NPPA and MZF1 mRNA levels in breast cancer patient. (d) The binding motif of MZF1. (e) The binding site of MZF1 to the promoter region of NPPA with PROMO and JASPAR. Binding sites in PROMO and JASPAR were labeled in red and blue, respectively. (f, g) Quantification of MZF1 and NPPA mRNA in MZF1 overexpressed MCF-7 (f) and MDA-MB-231 (g) cells. (h, i) ChIP result showing the binding of MZF1 to the -381~-452 bp of the NPPA promoter region in MZF1 overexpressed MCF-7 (h) and MDA-MB-231 (i) cells. (j, k) Dual luciferase assay showing the relative transcription activity of -300~-500 bp (Firefly/Renilla, RLU) in MZF1 overexpressed MCF-7 (j) and MDA-MB-231 (k) cells. Data are represented as mean ± SEM; ^∗^*p* < 0.05, ^∗∗^*p* < 0.01, and ^∗∗∗^*p* < 0.001. The data were analyzed using Student's *t*-test.

We further tried to explore the transcription modulation of NPPA in breast cancer cells. With PROMO and JASPAR, we predicted the binding of all human factors on the -1000 bp promoter region of NPPA. With dissimilarity < 5% in PROMO and relative profile score > 90% in JASPAR, we identified 11 mutual transcription factors with both methods (Figure 5(a)). Then, we estimated the Pearson correlation between the mRNA level of these genes with NPPA, respectively (Figure 5(b)), and we observed that only MZF1 is highly correlated with the NPPA (Figure 5(c)). Consistently, we identified the reduced MZF1 level in tumor tissues, especially in the basal subtype of breast cancer tissues (Suppl. Figure 5A-5B).

The binding motif of MZF1 is shown in Figure 5(d), and the corresponding binding site of MZF1 in PROMO and JASPAR was mainly clustered in the -750 bp and -350 bp regions of the NPPA promoter (Figure 5(e)). Consistently, we observed that the expression of NPPA was elevated in MCF-7 and MDA-MB-231 cells overexpressed with flag-tagged MZF1 (Figures 5(f) and 5(g)). To validate our findings, we explored the binding of MZF1 in the promoter region of NPPA with ChIP experiments. We observed the binding of MZF1 to the -318~-452 bp, but not the -889~-762 bp, of the NPPA promoter in both cell lines (Figures 5(h) and 5(i)). Further, we performed the dual luciferase assay with the -300~-500 bp of the NPPA promoter and observed a enhanced transcription activity of NPPA in MZF1 overexpressed MCF-7 and MDA-MB-231 cells (Figures 5(j) and 5(k)).

## 4. Discussion

Breast cancer is one of the leading cause of tumor-related death worldwide, whose incidence rate has been increasing for decades. While various approaches of treatment as well as multiple molecular subtypes of breast cancer have been proposed, the recurrence of breast cancer remains high and the biological significance of ECM-related genes, especially the function and role of NPPA in breast cancer patients, have not been discussed.

Here, in this study, we performed a novel SC3 clustering method of breast cancer patients with respect to the expression pattern of ECM-related genes and identified a cluster of breast cancer patients with much lower recurrence rate (Figure 1). We collected the marker genes for this cluster of breast cancer patients and assessed the correlation between the expression level of ECM-related genes and their prognosis with integrated bioinformatics analysis. We eventually identified NPPA as a novel prognostic marker for breast cancer patients. Also, NPPA remains a significant prognostic in breast cancer patients stratified by postoperative radiation condition, but not by ER status, PR status, and PAM50 subtypes.

The expression pattern of NPPA across various human tissues and cancer types has been demonstrated, and NPPA is simultaneously downregulated in cancer tissues. Further, we observed the reduced NPPA in mRNA and protein levels and the knockdown of NPPA result in the enhanced proliferation, migration, and invasion of breast cancer cells both in vivo and in vitro. However, the expression of NPPA in breast cancer is based on a computational study and further validation in patients is needed.

Eventually, we explored the transcription modulation of NPPA in breast cancer cells. We identified MZF1 as a key transcription factor for NPPA and predicted the binding of MZF1 to the two different domains of the NPPA promoter region. With the chromatin immunoprecipitation (ChIP) experiment as well as the following dual luciferase assay, we identified that the binding of MZF1 to the -300~-500 bp of the NPPA promoter could promote the transcription of NPPA.

Together, we plotted the expression pattern of ECM-related genes in breast cancer patients and identified a novel independent prognostic factor, NPPA, in the breast cancer patients. With both bioinformatics and experimental approaches, we established the expression pattern of NPPA and explored its biological function in breast cancer cells. We believe our findings would provide a novel insight into the prevention of breast cancer recurrence and provide a new approach in the treatment of breast cancer.

## Data Availability

Public datasets in the current study can be accessed from following links: TCGA BRCA database (https://tcga-data.nci.nih.gov/), Human Protein Atlas (https://www.proteinatlas.org/), GEPIA (http://gepia.cancer-pku.cn/index.html), GTEx database (https://xenabrowser.net/), PROMO database (http://alggen.lsi.upc.es/recerca/frame-recerca.html), and JASPAR database (http://jaspar.genereg.net/).

## Supplementary Materials

Supplementary Materials Supplementary Figure 1: (A) quantification of pathological stages, AJCC stages, and postoperative radiation therapy condition in Cluster 1 and Cluster 2 breast cancer patients. (B) Quantification of PR status and PAM50 subtypes in Cluster 1 and Cluster 2 breast cancer patients. (C) Quantification of postoperative new tumor events, new tumor event anatomic site, and new tumor event types in Cluster 1 and Cluster 2 breast cancer patients. Supplementary Figure 2: (A) Kaplan-Meier analysis comparing the prognostic value of ADAMTS13, SEMA3B, GDF15, EDIL3, MFAP4, and COL17A1 in breast cancer patients. DSS, DFI, and PFI were analyzed. (B–D) Kaplan-Meier analysis comparing the DSS and PFI of breast cancer patients with high or low NPPA level. Patients were stratified by ER status (B), PR status (C), or PAM50 subtypes (D). Supplementary Figure 3: (A–C) the expression pattern of NPPA in breast cancer patients with different postoperative radiation conditions (A), ER status (B), and AJCC staging (C). (D, E) Dot plot showing the correlation between NPPA mRNA and stemness score (D) and relative telomere length (E). (F) Dot plot showing the correlation between NPPA mRNA with the tumor infiltration T cell subtypes. The data were analyzed using Student's *t*-test, ^∗^*p* < 0.05. Supplementary Figure 4: (A) quantification of NPPA mRNA level in 4 NPPA depleted or control breast cancer cell lines. (B, C) Quantification of necrosis and apoptosis cell percentage with flowcytometry in NPPA depleted or control MCF-7 (B) and MDA-MB-231 (C) cells. (D, E) Quantification of body weight (D) and tumor weight (E) in nude mouse xenograft model with NPPA depleted or control MCF-7 cells. Data are represented as mean ± SEM, ^∗∗^*p* < 0.01. The data were analyzed using Student's *t*-test. Supplementary Figure 5: (A) the expression pattern of MZF1 in the TCGA breast cancer database. The data were analyzed using paired Student's *t*-test; ^*^*p* < 0.05. (B) The expression pattern of MZF1 in different subtypes of breast cancer in the GSE65216 database. The data were analyzed using Student's *t*-test; ^∗∗∗∗^*p* < 0.0001.
