# Supplementary material for: Integrated Bioinformatics and Experimental Approaches Identified the Role of NPPA in the Proliferation and the Malignant Behavior of Breast Cancer
Source: J Immunol Res. 2021 Sep 27;2021:7876489. doi: 10.1155/2021/7876489 (PMC8490067; doi:10.1155/2021/7876489)
Supplement: Supplementary Materials — Supplementary Figure 1: (A) quantification of pathological stages, AJCC stages, and postoperative radiation therapy condition in Cluster 1 and Cluster 2 breast cancer patients. (B) Quantification of PR status and PAM50 subtypes in Cluster 1 and Cluster 2 breast cancer patients. (C) Quantification of postoperative new tumor events, new tumor event anatomic site, and new tumor event types in Cluster 1 and Cluster 2 breast cancer patients. Supplementary Figure 2: (A) Kaplan-Meier analysis comparing the prognostic value of ADAMTS13, SEMA3B, GDF15, EDIL3, MFAP4, and COL17A1 in breast cancer patients. DSS, DFI, and PFI were analyzed. (B–D) Kaplan-Meier analysis comparing the DSS and PFI of breast cancer patients with high or low NPPA level. Patients were stratified by ER status (B), PR status (C), or PAM50 subtypes (D). Supplementary Figure 3: (A–C) the expression pattern of NPPA in breast cancer patients with different postoperative radiation conditions (A), ER status (B), and AJCC staging (C). (D, E) Dot plot showing the correlation between NPPA mRNA and stemness score (D) and relative telomere length (E). (F) Dot plot showing the correlation between NPPA mRNA with the tumor infiltration T cell subtypes. The data were analyzed using Student's t-test, ∗p < 0.05. Supplementary Figure 4: (A) quantification of NPPA mRNA level in 4 NPPA depleted or control breast cancer cell lines. (B, C) Quantification of necrosis and apoptosis cell percentage with flowcytometry in NPPA depleted or control MCF-7 (B) and MDA-MB-231 (C) cells. (D, E) Quantification of body weight (D) and tumor weight (E) in nude mouse xenograft model with NPPA depleted or control MCF-7 cells. Data are represented as mean ± SEM, ∗∗p < 0.01. The data were analyzed using Student's t-test. Supplementary Figure 5: (A) the expression pattern of MZF1 in the TCGA breast cancer database. The data were analyzed using paired Student's t-test; *p < 0.05. (B) The expression pattern of MZF1 in different subtypes of breast [file 7876489.f1.docx]

**Supplementary figure 1**


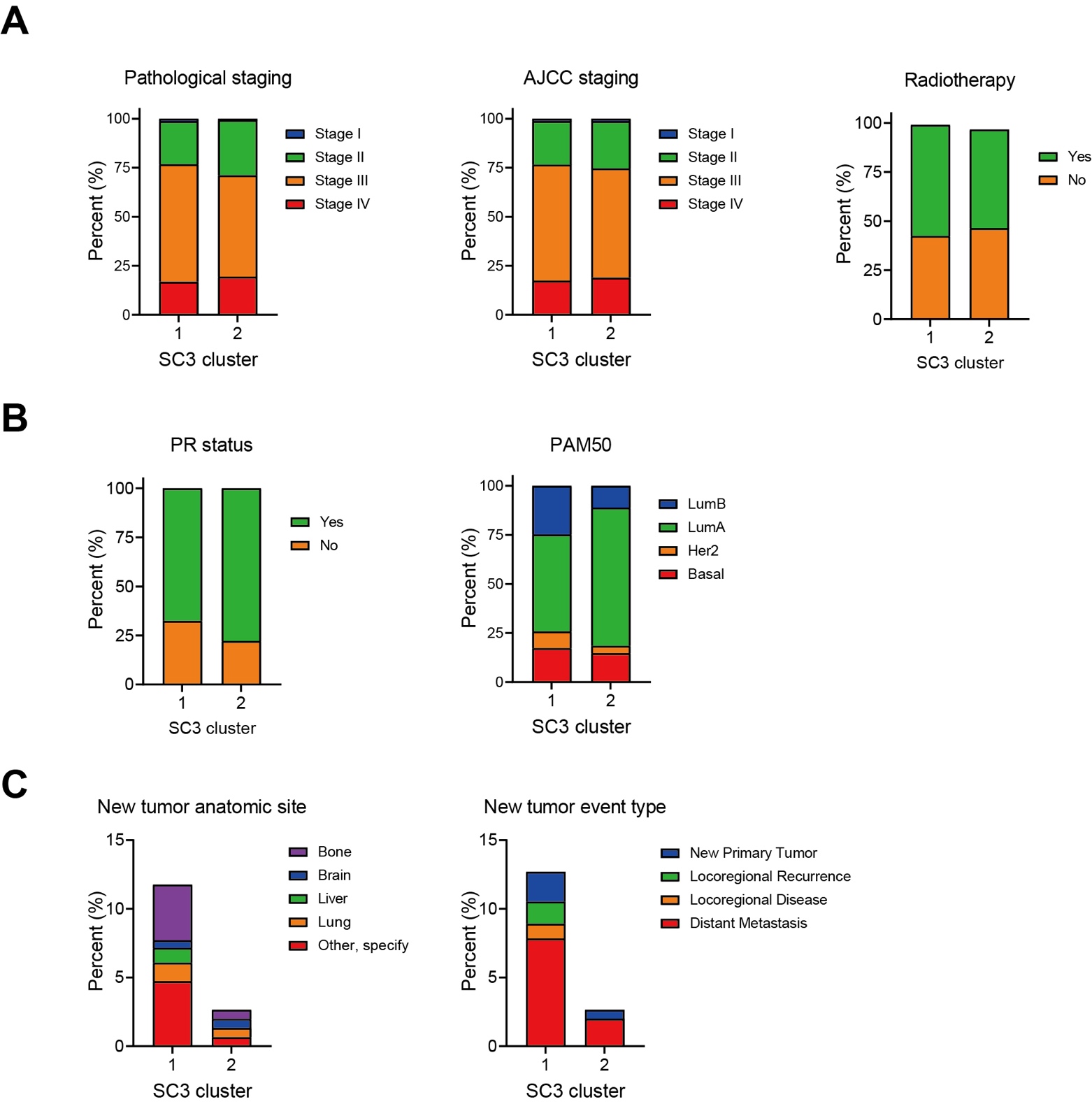


(A) Quantification of pathological stages, AJCC stages and postoperative radiation therapy condition in Cluster 1 and Cluster 2 breast cancer patients.

(B) Quantification of PR status and PAM50 subtypes in Cluster 1 and Cluster 2breast cancer patients.

(C) Quantification of postoperative new tumor events, new tumor event anatomic site and new tumor event types in Cluster 1 and Cluster 2 breast cancer patients.

**Supplementary figure 2**

**
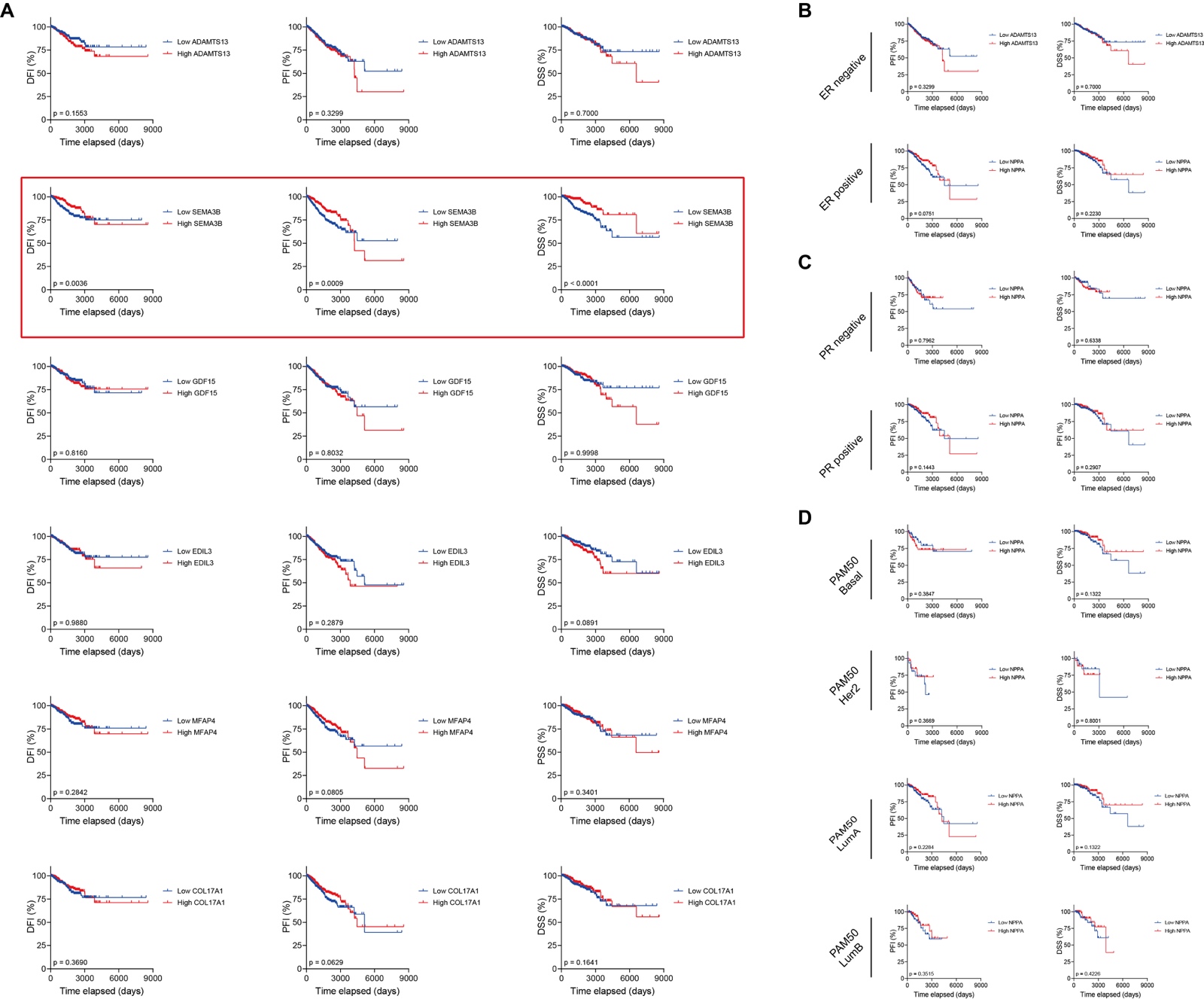
**

(A) Kaplan-Meier analysis comparing the prognostic value of ADAMTS13, SEMA3B, GDF15, EDIL3, MFAP4 and COL17A1 in breast cancer patients. DSS, DFI and PFI were analyzed.

(B-D) Kaplan-Meier analysis comparing the DSS and PFI of breast cancer patients with high or low NPPA level. Patients were stratified by ER status (B), PR status (C) or PAM50 subtypes (D).

**Supplementary figure 3**


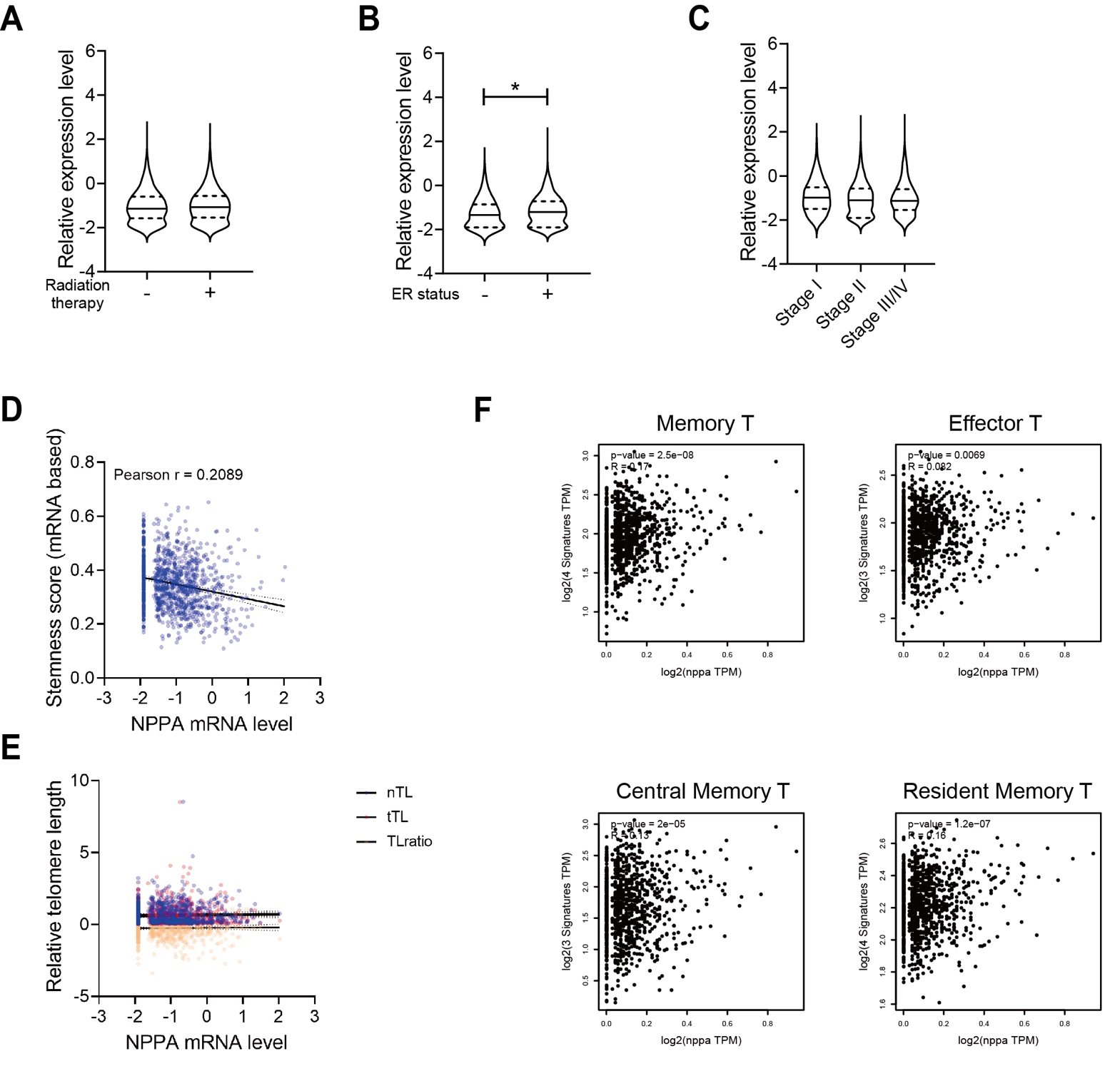


(A-C) The expression pattern of NPPA in breast cancer patients with different postoperative radiation condition (A), ER status (B) and AJCC staging (C).

(D-E) Dot plot showing the correlation between NPPA mRNA and stemness score (D) and relative telomere length (E).

(F) Dot plot showing the correlation between NPPA mRNA with the tumor infiltration T cell subtypes.

**Supplementary figure 4**


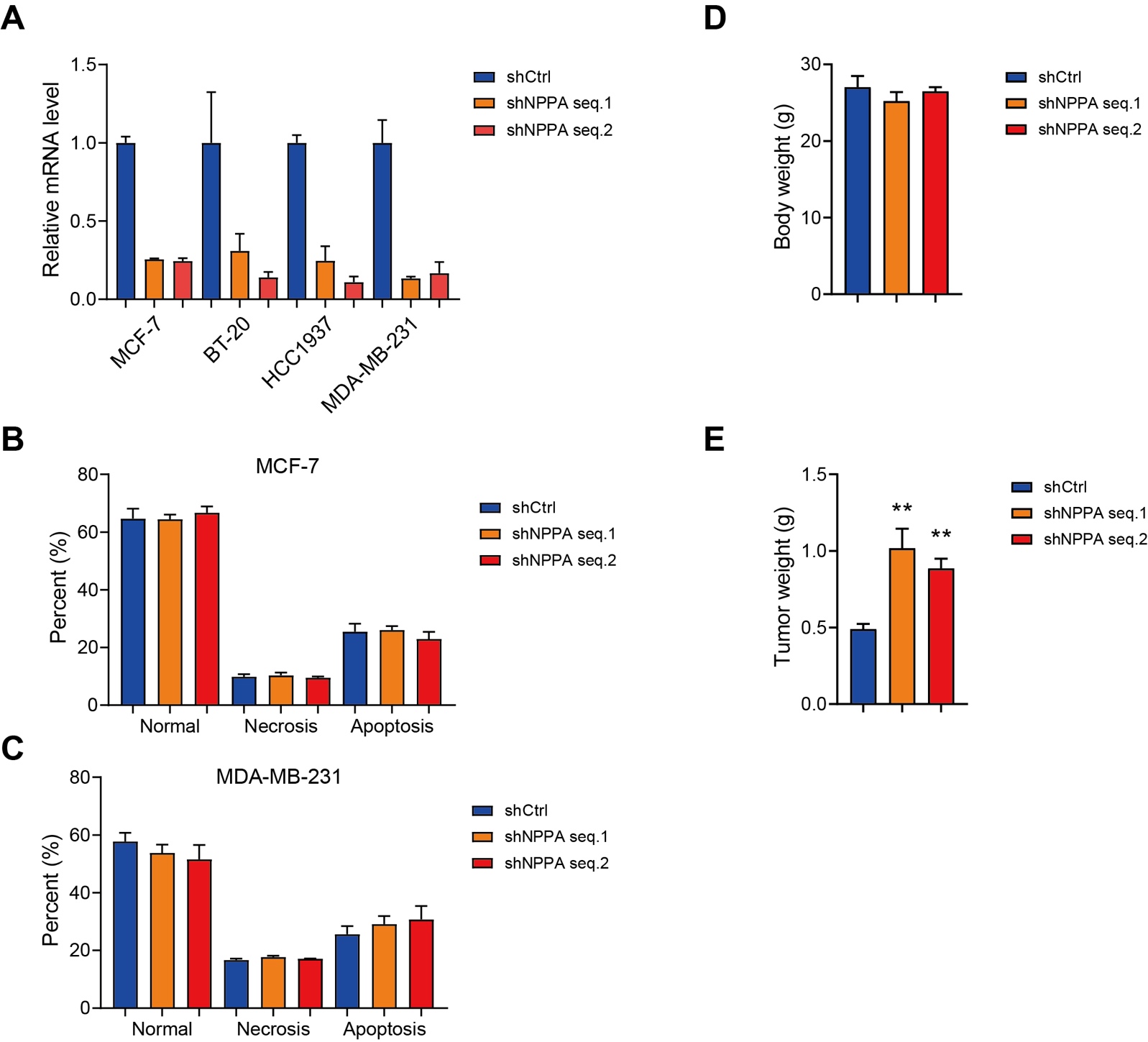


(A) Quantification of NPPA mRNA level in 4 NPPA depleted or control breast cancer cell liens.

(B-C) Quantification of necrosis and apoptosis cell percentage with flowcytometry in NPPA depleted or control MCF-7 (B) and MDA-MB-231 (C) cells.

(D-E) Quantification of body weight (D) and tumor weight (E) in nude mice xenograft model with NPPA depleted or control MCF-7 cells.

**Supplementary figure 5**


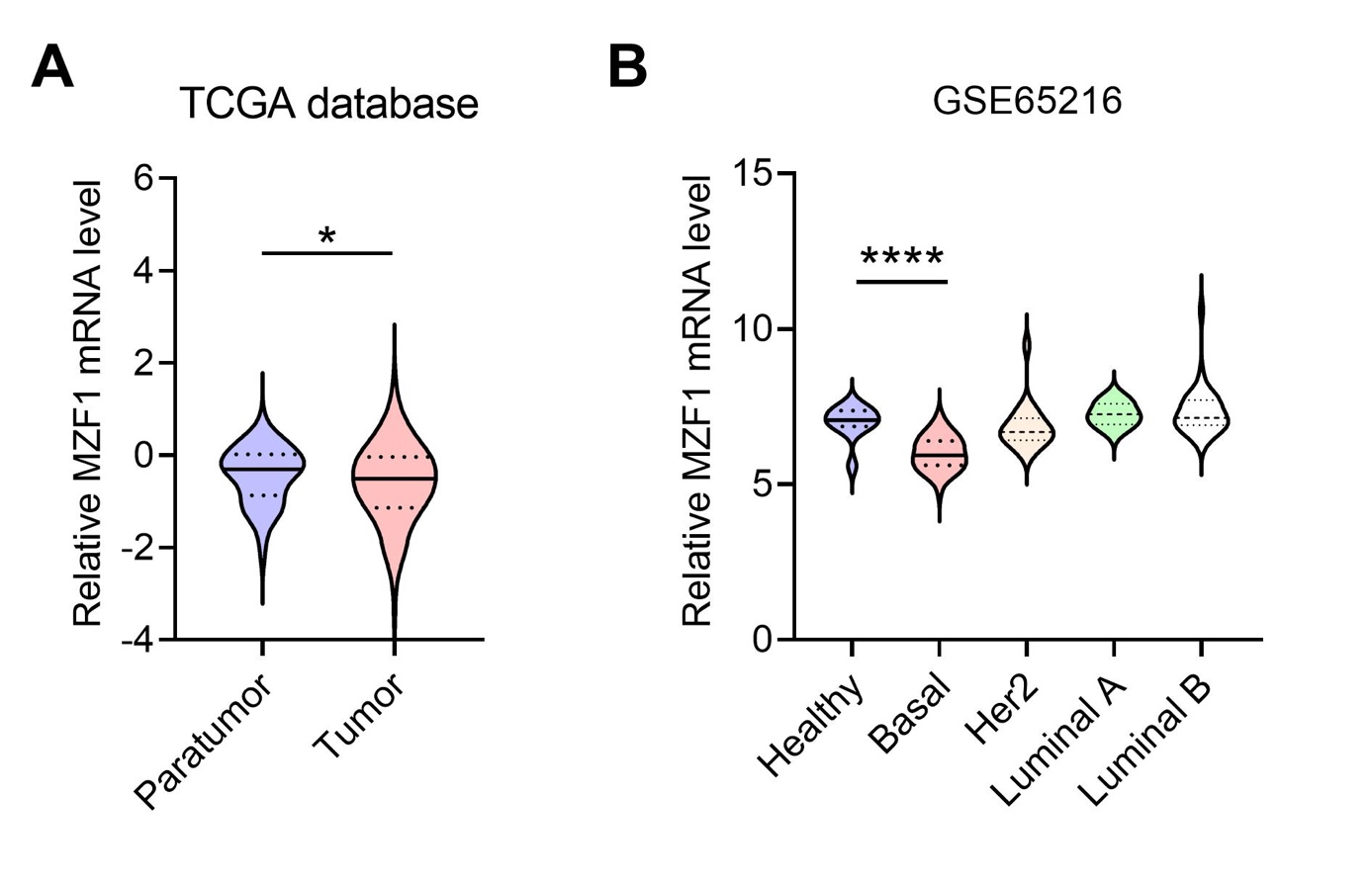


(A) The expression pattern of MZF1 in TCGA breast cancer database. The data were analyzed using paired Students’ *t* test; * p < 0.05.

(B) The expression pattern of MZF1 in different subtypes of breast cancer in GSE65216 database. The data were analyzed using Students’ *t* test; **** p < 0.0001.
